# Strategies and outcomes of different methods for treating abdominal aortic stent graft infection

**DOI:** 10.3389/fcvm.2023.1180050

**Published:** 2023-08-07

**Authors:** Mengqiang Zhang, Zhipeng Chen, Chen Tang, Cheng Liu, Xiaoqiang Li, Zhao Liu, Tong Qiao

**Affiliations:** Department of Vascular Surgery, Affiliated Drum Tower Hospital, Medical School of Nanjing University, Nanjing, China

**Keywords:** abdominal aortic aneurysm, endovascular aortic repair, stent graft, infection, treatment

## Abstract

**Objective:**

To report the strategies and short-term results of different treatment methods for abdominal aortic stent graft infection.

**Methods:**

Six consecutive patients (5 males and 1 female; mean age: 64 years; age range: 49–79 years) received surgical treatment for stent graft infection from November 2021 to December 2022. All patients underwent endovascular aortic repair (EVAR) for abdominal aortic and iliac artery disease, subsequently developed graft infection and then received corresponding surgical treatment with different materials (artificial blood vessel, bovine pericardium, autologous great saphenous vein) in our department. The outcomes were analysed.

**Results:**

Immediate technical success was achieved intraoperatively in all six patients. The 30-day mortality rate was 0%. During a mean follow-up of 4 months (range, 3–13 months), one patient underwent a second operation due to vascular anastomotic haemorrhage and underwent bilateral limb amputations due to ischaemia. All patients survived.

**Conclusions:**

In the short term, the different materils and methods used to treat aortic stent graft infection achieved satisfactory results.

## 1. Introduction

The treatment of abdominal aortic aneurysm has experienced a transformation from traditional open repair to minimally invasive endovascular repair (EVAR) in select cases. EVAR can reduce the short-term mortality of patients compared with open surgery, but there is no significant difference in the long-term mortality between the two methods (1). Moreover, EVAR can also lead to serious complications, such as aortic stent-graft infection (SGI), which can be fatal for patients. According to reports in the literature, the incidence of SGI is 0.2%–30% (2–6). Surgical approaches (taking out the infected stent and surrounding tissue and then revascularization) combined with long-term anti-infective treatment before and after surgery is a relatively recommended treatment method at present. However, there are no standardized guidelines for the timing of the surgery, the mode of revascularization, or the materials used in such patients. This study reports our medical centre's experience with treating SGI with different materials.

## 2. Materials and methods

### 2.1. Patient selection, characteristics, and definitions

**Figure 1:**
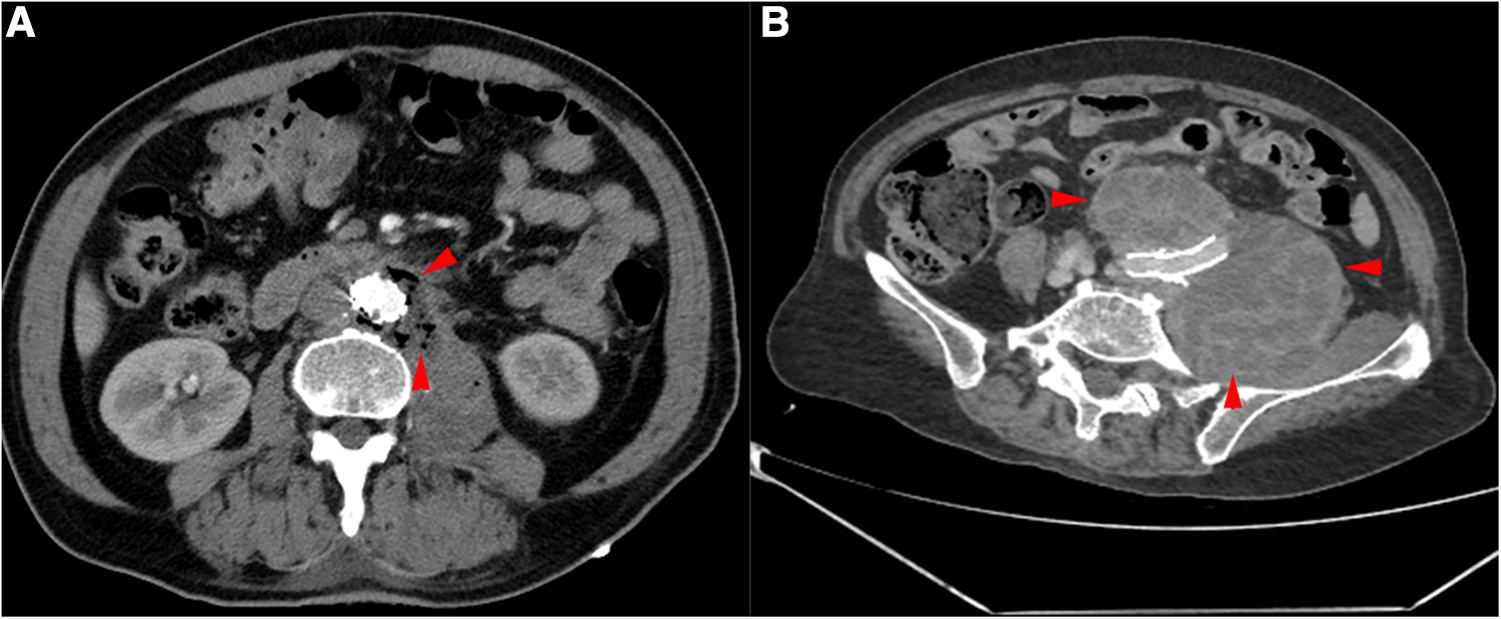
CTA of the patient's aorta. (**A**) Red arrows indicate the presence of gas around the aorta. (**B**) Red arrows indicate haematoma around the common iliac artery.

**Figure 2:**
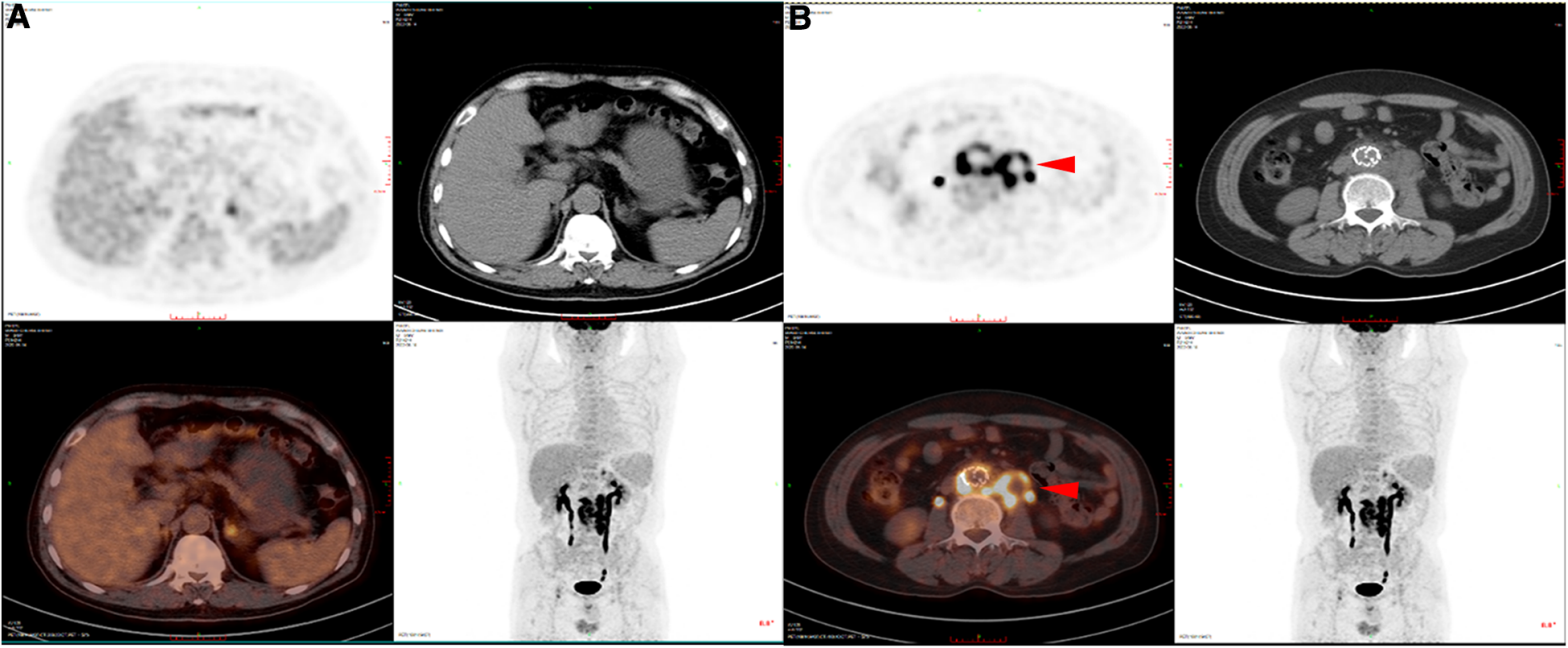
PET-CT of SGI patients. (**A**) PET-CT images of non-infected sites showed no obvious fluorescence enhancement. (**B**) PET-CT images of the infected sites showed obvious fluorescence enhancement.

This retrospective study was approved by the Ethics Committee of Nanjing Drum Tower Hospital (registration number: 2017-015-05), and all patients provided consent for their participation. Between November 2021 and December 2022, patients with SGI were treated with removal of the infected grafts and surrounding infected tissues and revascularization (five men, one woman; mean age, 64 years; range, 49–79 years). Prior to identification of the specific infection and sensitive antibiotics, patients were treated with empiric broad-spectrum antibiotics, and surgical treatment was performed when the patient's clinical symptoms were relieved (abdominal pain, elevated body temperature and presence of inflammatory indicators). If the patient's condition worsened, emergency surgery was considered. All patients presented with postoperative fever or pain after EVAR (persistent fever in five patients and pain in the left lower abdomen in one patient). Currently, the diagnosis of SGI relies mainly on imaging, clinical presentation and laboratory indicators. The patients commonly presented with postoperative fever, and the imaging results typically showed an annular low-density shadow around the aorta, gas, and multiple floc shadows and lymph node shadows (with partial enlargement) (Figure 1A,B); the relevant inflammatory indicators (leukocytes, neutrophils, interleukin-6) were significantly increased. Aortic CT angiography (CTA) was commonly used, as was positron emission tomography/computed tomography (PET-CT). For some more insidious lesions, PET-CT has obvious advantages. PET-CT perfectly integrates PET and CT. PET provides detailed functional and metabolic molecular information of the lesion, while CT provides precise anatomical location of the lesion. Thus, PET-CT can achieve early detection of lesions and diagnosis of diseases (the radionuclide we used was 18F-FDG) (Figure 2A,B). Fever was defined as an axillary temperature above 38.0°C. The patient characteristics are summarized in the Table.

### 2.2. Procedure details

#### 2.2.1. Removal of the original abdominal aortic stent

**Figure 3:**
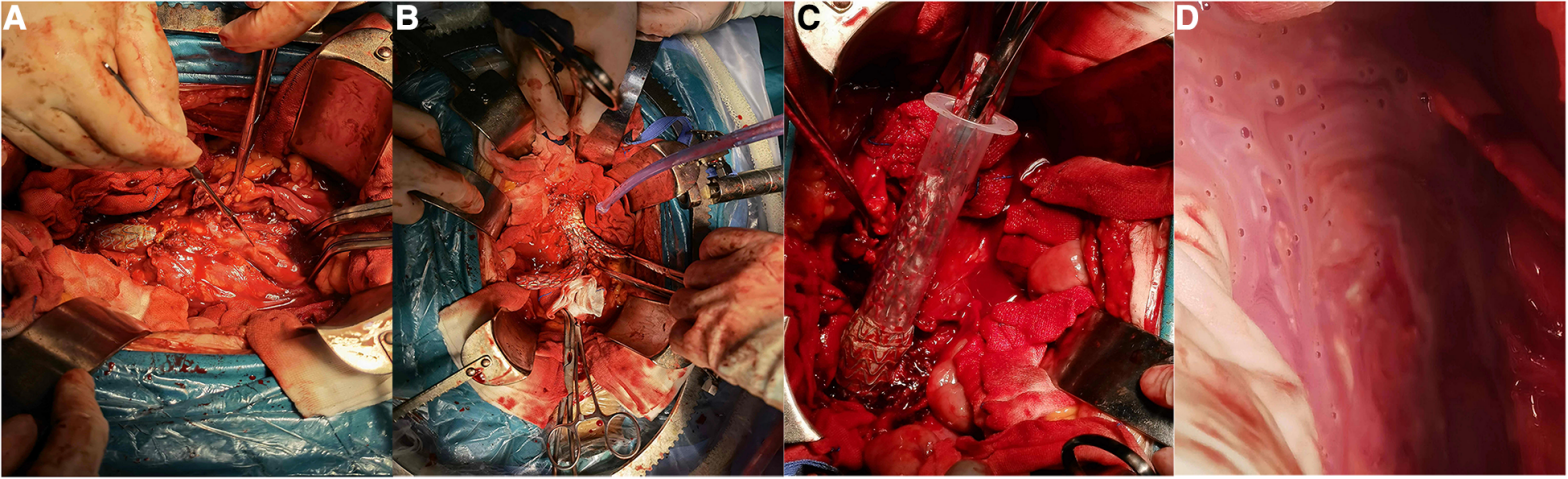
Intraoperative images of SGI patients. (**A**) The right iliac artery was dissected before stent exposure. (**B**) Abdominal aortic stents and bilateral iliac artery stents were exposed. (**C**) Because the stent was barbed, the 20 ml syringe was used to remove the stent. (**D**) Purulent fluid around the iliac artery.

After dissecting the abdominal aortic aneurysm cavity, purulent secretions (preserved for culture) and covered stents could be seen. Because the abdominal aortic stent has a barbed structure, a 20 ml syringe was trimmed, and the abdominal aortic stent was cut off after blocking the blood flow at the proximal end of the abdominal aorta; then, the syringe was used to recover the proximal stent and extract the original iliac artery stent from the distal end. Large quantities of iodophor and hydrogen peroxide were used to flush the infected area, and the abdominal incision was closed after retaining the drainage tube (Figure 3A–D).

#### 2.2.2. Axillary-femoral artery and femoral-femoral artery bypass grafting

The right axillary and femoral arteries were dissected and exposed, and subcutaneous tunnels were established. Artificial blood vessels (8 mm Gore, USA) were used for end-to-side anastomosis with the axillary artery and femoral artery, respectively. Femoral artery-femoral artery bypass was achieved using an artificial vessel or an autogenous great saphenous vein.

#### 2.2.3. Biological mesh - bovine pericardial reconstruction *in situ*

**Figure 4:**
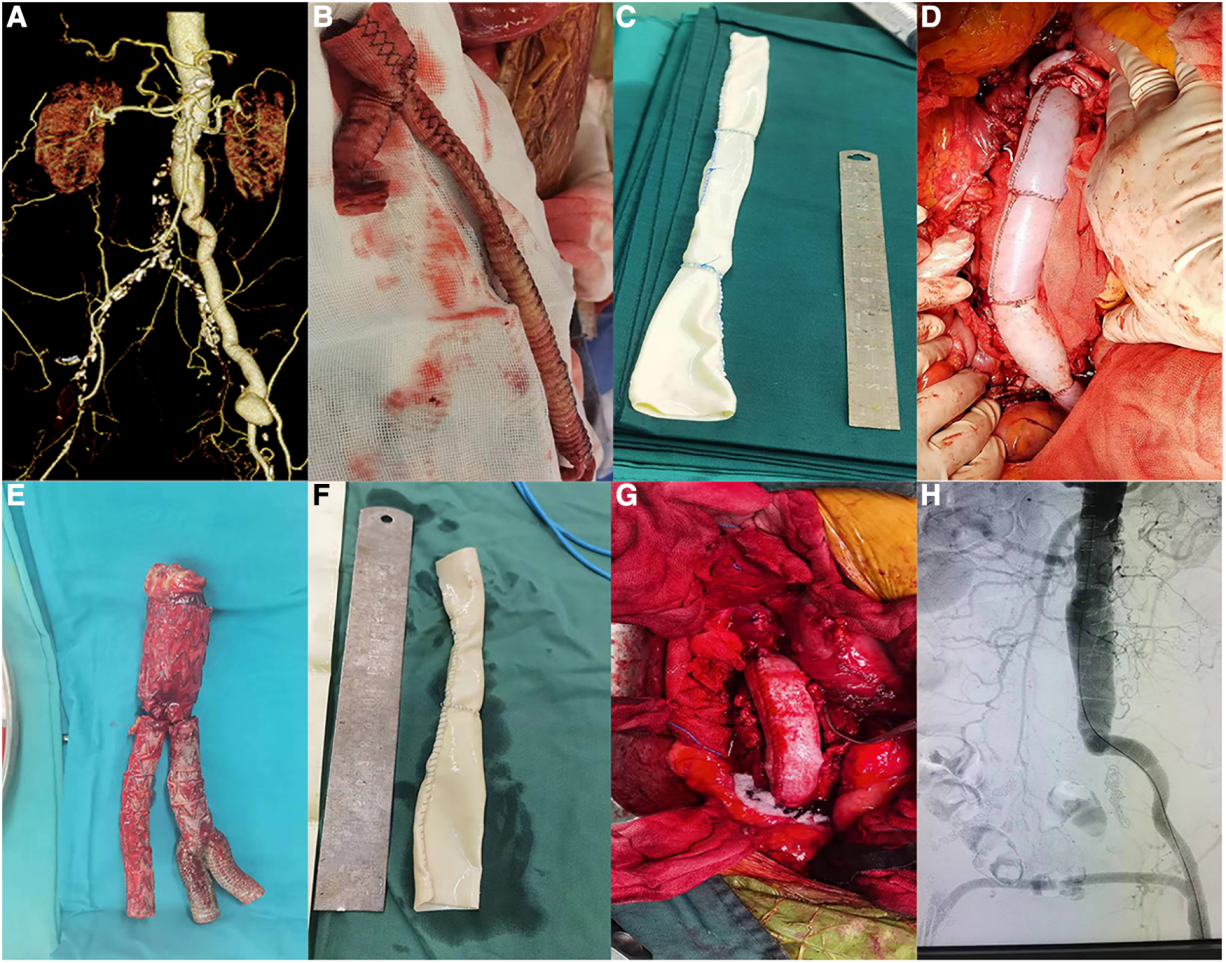
Photos of patients undergoing surgery (patients 4 and 5). (**A**) Preoperative CTA of patient 4. (**B**) Removal of the artificial blood vessel during surgery (patient 4). (**C**) Sutured bovine pericardium (patient 4). (**D**) Bovine pericardium anastomosed with the patient (patient 4). (**E**) Stents removed during surgery (patient 5). (**F**) Sutured bovine pericardium (patient 5). (**G**) Bovine pericardium anastomosed with the aorta (patient 5). (**H**) Postoperative aortogram image (patient 5).

The transplanted blood vessel was sewn with Bovine pericardial (10.5 cm × 10.0 cm, Balance Medical, China). After trimming to the patch to the appropriate length, the proximal end of the biograft was anastomosed end-to-end with the abdominal aorta, and the distal end was anastomosed end-to-end with the left common ilium. Then, the opening in the right iliac artery was closed (Figure 4A–H).

### 2.3. Statistical analysis

Excel software was used to describe the data. Quantitative data were expressed as median (range), and categorical data were expressed as frequency.

## 3. Results

### 3.1. Intraoperative outcomes and early results (30 days)

**Figure 5:**
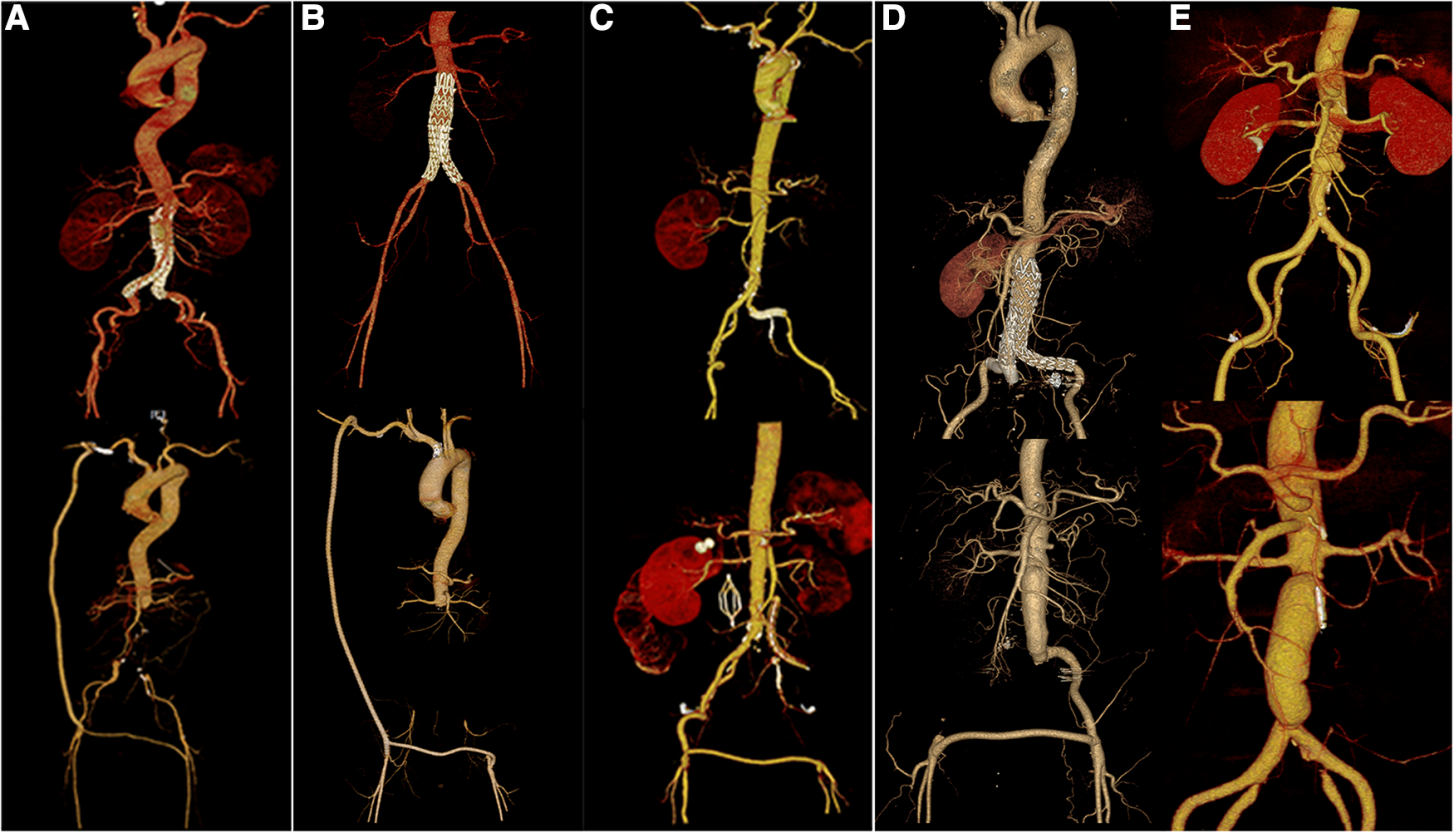
(**A–E**). Preoperative and postoperative CTA data of patients 1–3, 5, and 6, respectively.

Immediate technical success was achieved intraoperatively in all patients (Figure 5A–E). The mean procedure time was 505 min (range, 370–655 min). All patients underwent autologous whole-blood resuscitation. All patients were transferred to the ICU for further treatment after surgery because of extensive trauma, the long surgical duration and excessive intraoperative bleeding. In these patients, the infecting microorganisms detected by ascites assay included Proteus Mirmiris, Klebsiella pneumoniae, and Staphylococcus epidermidis. Acinetobacter baumannii, Staphylococcus epidermidis, Staphylococcus haemolyticus, Candida albicans, and Morganella were found in blood cultures. All patients received anti-infective therapy with sensitive antibiotics.

**Figure 6:**
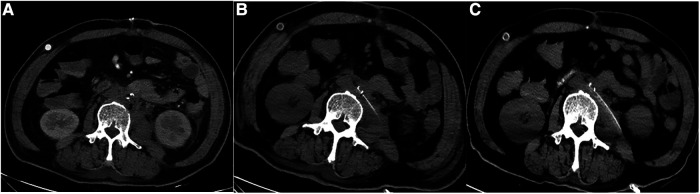
Abdominal CT was repeated at different points after the operation (patient 1). (**A**)Abdominal aortic CT at 1 week after operation showed that there was still a small amount of gas in the stump. (**B**) Abdominal aortic CT at 2 weeks after operation showed that the residual air was further reduced. (**C**) Abdominal aortic CT at 1 month after operation showed that the residual air basically disappeared.

Among the patients, patient 1 had a complex illness and spent 44 days and 8 h in the ICU. Patient 1 visited the local hospital due to abdominal pain before the first stent implantation, and the aortic CTA showed penetrating ulcer and exudation around the abdominal aorta (rupture could not be excluded), so the local hospital gave the patient abdominal aortic stent implantation, and he developed fever more than 1 month after the operation. The secretions were infected with gram-negative bacteria (Klebsiella pneumoniae, Proteus mirabilis), which were sensitive to Tienam. As a result, the patient was given anti-infective treatment. After the fever was brought under control, the abdominal aorta was resected with an artificial vascular bypass. After 10 days in the ICU, the patient had stable circulation, clear consciousness, and strong spontaneous breathing. After passing the spontaneous breathing test (SBT), the tracheal catheter was removed. The patient, however, continued to exhibit intermittent fever. Examination with abdominal CT (2022-08-22) indicated multiple effusions with a small amount of gas around the abdominal aorta (Figure 6A). The patient then underwent CT-guided puncture drainage, and the drainage fluid was sent for culture. Meanwhile, the patient's anaemia failed to be corrected with a transfusion, and ulcer bleeding was detected by gastroscopy and treated simultaneously. After a week (2022-08-30), abdominal CT showed less gas and fluid accumulation around the abdominal aorta (Figure 6B). A second abdominal CT scan was performed half a month later (2022-09-14), and the results showed that the accumulation of gas and fluid around the abdominal aorta was further reduced (Figure 6C). During the patient's ICU stay, however, the blood culture suggested bacterial (Staphylococcus epidermidis, Staphylococcus haemolyticus) and Candida albicans infection, and the patient was consistently given antibiotics.

Patient 4 was admitted to hospital with a pulsating mass in his left groin that had been present for more than seven years and was accompanied by a localised black coloration. In 2013, the patient underwent aortic-bilateral femoral bypass grafting, a left femoral endarterectomy, and a left lower extremity embolism due to aortic-iliac occlusion and left lower extremity embolism at another hospital. The bilateral inguinal infection developed after the operation. After admission, the patient had a palpable pulsatile mass in the left inguinal region with dark skin color, a sinus tract about 2 cm × 2 cm in the right inguinal region, and ischemia in the right lower limb (right toe cyanosis). Bacterial cultures from two consecutive days of admission showed no significant bacteria. PET-CT examination revealed persistent pseudoluminal and soft tissue thickening around the lumen of the abdominal aorta, the bilateral common iliac artery and the bilateral external iliac artery. At the time, it was thought that pseudaneurysms with infection were highly likely. The operation of “aortic prosthesis resection, left femoral artery pseudoaneurysm resection, abdominal aorto-left femoral artery reconstruction with biological materials, right inguinal debridement, right lower limb artificial blood vessel exit, ureteral anastomosis, and Double J tube” was carried out. Due to difficulty in maintaining patient 4’s postoperative blood pressure, an abdominal CT examination was performed, and the result indicated bleeding at the abdominal aortic anastomosis, so the patient underwent a second surgical procedure (retroperitoneal haematoma removal and abdominal aortic anastomosis). Due to the poor blood supply to the right lower limb, the patient underwent amputation of the right lower limb after one week.

Patient 6 developed a high fever (bacteraemia) again after surgery and was transferred to the Department of Infectious Diseases of our hospital for continued treatment. After more than a month of anti-infection treatment, the patient's condition improved, and he was switched to oral antibiotics after discharge.

### 3.2. Follow-up

At a mean follow-up of 175 days (range, 81–395 d), there has been no deaths, and the surgical incisions had healed well. Patient 4 had his left lower leg amputated at a local hospital. Additionally, Patient 1 and Patient 2 complained of discomfort in their lower extremities after walking for approximately 1,000 m and needed rest. This may be related to the unsatisfactory blood supply to the lower extremities in these patients.

## 4. Discussion

In the current medical context, where minimally invasive methods are increasingly being pursued, there will be a greater focus on the development and application of stents. SGI is an extremely complex clinical challenge that is likely to be encountered more frequently in the future. The specific cause of this disease is unclear and is reported to be related to the following factors: 1. worse aseptic conditions in the interventional theatre than in the operating theatre; 2. bacterial colonization caused by increased adhesion of the scaffold grafts; 3. aortic fistula due to mechanical erosion; 4. persistent blood clots in the aneurysm that can increase bacterial colonization; and 5. wound-associated infection or distant infection site (lung, urethra) (7, 8). SGI is sometimes difficult to diagnose clinically. The Management of Aortic Graft Infection Collaboration (MAGIC) provides reference suggestions for the diagnosis of SGI (9). The diagnostic criteria are divided into major and minor criteria, in which the major criteria include the intraoperative identification of pus surrounding a graft and the presence of direct communication between the prosthesis and a nonsterile site, including fistulae, exposed grafts in open wounds, and deployment of an endovascular stent-graft into an infected field (e.g., mycotic aneurysm); increasing perigraft gas volume on serial CT imaging or perigraft gas or fluid (≥7 weeks and ≥3 months, respectively) postimplantation; and isolation of microorganisms from percutaneous aspirates of perigraft fluid, explanted grafts, and other intraoperative specimens. The minor criteria include fever ≥38°C with SGI as the most likely cause; a positive blood culture or elevated inflammatory index with no alternative source; and other CT features or evidence from alternative imaging techniques. It has been proposed that SGI should be suspected if a single major criterion or two or more minor criteria from different classes are present. SGI is diagnosed if one major criterion plus any other criterion (major or minor) from the same category are met. Of the three, lab tests play an extremely important role. First, if preoperative inflammatory indicators are controlled to the desired range, this means that the patient is likely to have a better prognosis, which leaves room for clinicians to choose the timing of surgery. Postoperative inflammatory indicators can also predict the effect of surgery. If the indicators are good, that will be a huge psychological comfort for doctors.In daily clinical work, however, when the patient has no obvious clinical symptoms (such as fever) or typical imaging findings, it is very difficult for clinicians to identify SGI in the early stage. This is also a major factor in the poor outcomes of such patients.

This study evaluated six patients with SGI treated with different materials (artificial blood vessel, bovine pericardium, autologous great saphenous vein). Regardless of the material, the operation was successful. Although Patient 4 underwent multiple operations, this may have been due to the patient's poor health, which had little to do with the materials used in the operations. We can also see from the length of the procedure and the length and cost of the stay that this disease is not only a huge challenge for doctors but also a huge burden to patients. This kind of operation has high requirements for the operator. Fortunately, none of our patients died during follow-up, which shows to some extent that our treatment was relatively successful. There are two surgical options for aorta reconstruction: *in situ* reconstruction [mainly with an antibiotic-soaked graft, silver graft, bovine pericardial tube, cryopreserved arterial allograft, or neo-aortoiliac system (NAIS) with femoropopliteal veins] and extra-anatomic reconstruction (10). In this study, for reconstruction of the abdominal aorta, artificial blood vessels were used for extra-anatomic reconstruction. In situ reconstruction was performed using the surgical biomaterial bovine pericardium. For the reconstruction between the femoral arteries, two materials are also used: one is artificial blood vessels, and the other is autogenous great saphenous veins. Currently, there is great controversy over which material should be used in surgery. Different surgical materials have their respective advantages. For bovine pericardium, the biological material has the following advantages: good patency, low reinfection rate, acceptably low early mortality rate, relatively low cost, versatile applicability and immediate availability (11, 12). For the purpose of preventing reinfection, extra-anatomic reconstruction is a commonly used method, but it also has some disadvantages, such as a low long-term patency rate (5-year 40%–73%), aortic stump rupture (20%), amputation (20%–29%), and reinfection (20%) (13, 14). Furthermore, the 30-day mortality rate in our study was 0%, which is lower than the 8%–36% reported in previous literature (15–17). This may be related to the small number of cases in our study. Argyriou and his colleagues reported that the 30-day mortality rate for SGI with conservative treatment was 63.3%, which was significantly higher than that for surgical treatment (18). As a result, the current clinical treatment of SGI is dominated by anti-infective therapy in combination with surgical treatment. Not only is the short-term prognosis poor, the long-term prognosis is even worse. According to the literature, even if the best conservative treatment is adopted, the 5-year mortality rate still reaches 50% (19–21). Therefore, from the point of view of current treatment principles, active surgical treatment is also beneficial for patients in the future.

Anti-infective treatment is generally maintained long term for these patients, or even lifelong, and sensitive antibiotics can be selected based on secretion culture or blood culture findings (15, 16). Some scholars suggest that antibiotics should be stopped only after all signs of infection have disappeared and blood bacterial culture results have turned negative, but some scholars recommend lifelong anti-infective treatment (22, 23). The patients continued to receive anti-infection treatment (intravenous drip or oral antibiotic) after discharge, and the duration until drug withdrawal was determined by the chief physician according to the patient's condition. This process reflects the importance of multidisciplinary collaboration. The surgical treatment for these patients may be only the beginning of the treatment plan. These patients are also at risk for reinfection, ischaemia of some organs and other complications after surgery. Treatment also requires cooperation from the operating room, anaesthesia department, ICU, infection department and others to help patients overcome difficulties.

The main limitations of our present study are as follows: 1. the sample size is small, which may be mainly due to the low incidence of the disease and the collection of case data from a single centre; and 2. the study is a retrospective study, and there may be biases in the data collection process. As the number of patients increases, we can carry out more meaningful research. The treatment of such patients will also become more standardized.

In summary, the treatment of SGI with different materials, with both *in situ* reconstruction and extra-anatomic reconstruction, is satisfactory in the short term.

## Data Availability

The original contributions presented in the study are included in the article/Supplementary Material, further inquiries can be directed to the corresponding authors.

## Supplementary material

Supplementary Table 1 Summary of patient characteristics and results of surgical treatment for SGI.
