# Supplementary material for: Strategies and outcomes of different methods for treating abdominal aortic stent graft infection
Source: Front Cardiovasc Med. 2023 Aug 7;10:1180050. doi: 10.3389/fcvm.2023.1180050 (PMC10441108; doi:10.3389/fcvm.2023.1180050)
Supplement: Supplementary Table 1 — Summary of patient characteristics and results of surgical treatment for SGI. [file Table1.doc]

| No. | 1 | 2 | 3 | 4 | 5 | 6 |
| --- | --- | --- | --- | --- | --- | --- |
| Age (y) | 70 | 49 | 67 | 79 | 53 | 64 |
| Sex | M | M | F | M | M | M |
| Diagnosis | AAA | AAD | Iliac aneurysm | Aortic - iliac artery occlusion | AAA | Abdomi-nal aortic ulcer with infection |
| Basic diseases | Diabetes | Hyperte-nsion | Diabet-es;DVT | Hyperte-nsion | Diabetes | Diabetes;Hyperte-nsion;ba-cteremia;Malignant tumor of colon |
| Time of initial stent implantation | 2022.06 | 2022.05.20 | 2022.05.26 | 2013.12 | 2019.03 | - |
| Time of surgical removal of the infected stent | 2022.08.08 | 2022.07.13 | 2022.09.20 | 2022.08.25 | 2021.11.10 | 2022.05.20 |
| Length of operation | 9 h | 10h55m-in | 6h20 | 10h45m-in | 6h10 | 7h20 |
| Intraoperative blood loss(ml) | 5000 | 5000 | 2300 | 4000 | 2000 | 2000 |
| Hospitalization days | 59 | 17 | 17 | 31 | 23 | 49 |
| ICU days | 46 | 6 | 5 | 20 | 5 | 11 |
| Ventilator use days | 9 | 2 | 4 | 5 | 2 | 6 |
| Revascularization material | Gore | Gore | Gore | bovine pericard-  ium | bovine pericardi-um | bovine pericardi-um |

AAA=abdominal aortic aneurysm, AD=aortic dissection, M=male, F=female
